# Subcutaneous Infiltration of Indocyanine Green From a Malpositioned Intravenous Catheter

**DOI:** 10.7759/cureus.16378

**Published:** 2021-07-13

**Authors:** Kevin Sigley, Pial Hope, Raymond Laird

**Affiliations:** 1 General Surgery, Beaumont Health, Dearborn, USA; 2 Surgery, Beaumont Health, Dearborn, USA; 3 Surgery, Beaumont Health, Trenton, USA

**Keywords:** indocyanine green, icg, firefly, infiltrate, extravasate, iv, infiltration, extravasation, da vinci, robot

## Abstract

Indocyanine green (ICG) is a water-soluble, iodine-containing molecule with a wide variety of applications in various fields of medicine. In this paper, we report an adverse event of ICG infiltration into subcutaneous tissue from a malpositioned intravenous (IV) catheter. Although ICG can be injected intradermally and subcutaneously for sentinel lymph node biopsy in breast cancer surgery, no reports exist regarding inadvertent infiltration from an IV catheter. It is our objective to provide an example should this unfortunate event occur in other populations, to describe the timing of resolution from infiltration, and to provide recommendations for future occurrences. In this case, the discoloration from infiltration became apparent on postoperative day one and had resolved completely at the time of the follow-up appointment on postoperative day 18.

## Introduction

Indocyanine green (ICG) is a water-soluble, iodine-containing, albumin-bound dye with a wide range of diagnostic medical applications, and has been in clinical use since the 1950s [1]. ICG binds to plasma lipoproteins if injected intravenously and provides insight regarding tissue perfusion by fluorescing when excited by near-infrared light. If injected into tissues, it migrates in the lymphatic system, enabling the identification of lymphatic basins [2]. ICG has been used in the fields of cardiology, ophthalmology, neurosurgery [3,4], hepatology, and oncology [1].

ICG fluoresces at about 800 nm [5]. Specifically designed laparoscopic and robotic cameras have modes for emitting wavelengths of light to excite ICG molecules and a near-infrared filter to record the fluorescence. Although ICG has many potential uses, our practice primarily uses it to delineate biliary anatomy as well as to investigate intestinal and anastomotic integrity.

In terms of intravenous (IV) catheter malfunction, extravasation refers to leakage of a vesicant medication or fluid (e.g., calcium chloride or gluconate, chemotherapeutic agents, vasopressors, among others) whereas infiltration refers to subcutaneous administration of a nonvesicant fluid or medication.

We present a case of ICG infiltration into a patient’s subcutaneous tissues due to a malpositioned IV catheter. Although ICG has been utilized to study the extravasation of chemotherapy and to map sentinel lymph nodes in breast cancer surgery [6,7], no prior case reports exist describing unintentional infiltration from an IV. We present this case to outline the expected clinical course of infiltrated subcutaneous ICG.

## Case presentation

A 59-year-old male with a history of perforated sigmoid diverticulitis managed with exploratory laparotomy and sigmoid resection with end colostomy (Hartmann procedure) nine months prior presented to our clinic to discuss colostomy reversal. A CT scan was obtained for preoperative planning and revealed a nonobstructive parastomal hernia containing small bowel. He was scheduled for a robot-assisted laparoscopic colostomy reversal and closure of the hernia defect.

Bilateral 18 gauge forearm IVs were placed preoperatively without reported adverse events on the day of the patient’s operation. He was taken to the operating room and an ultrasound-guided transversus abdominus block was performed. He was placed in a lithotomy position with both arms tucked at his sides, and a Foley catheter was inserted. The abdomen was accessed through the left upper quadrant with a spring-loaded needle, pneumoperitoneum was achieved, and four 8 mm robotic trocars were placed. The patient was placed in the Trendelenburg position to aid in the pelvic dissection. The intraperitoneal segment of the colostomy was dissected free and mobilized proximally along the white line of Toldt to ensure a tension-free anastomosis to the rectal stump. Next, the robot was undocked and an elliptical skin incision was made around the colostomy.

The incision was carried down into the subcutaneous tissues to the level of the hernia sac, which was dissected free from the underlying fascia. A wound protector was placed and the colon was extracorporealized. Once an adequate site of resection was determined, the mesocolon was dissected free from the colon and transected. A 29 mm end-to-end anastomosis (EEA) stapler was chosen to fashion the anastomosis, and the anvil was secured to the transected colon with braided absorbable suture in purse-string fashion. The colon with anvil was delivered back into the peritoneal cavity and the wound protector cover was attached. The abdomen was re-insufflated and the patient was placed in the Trendelenburg position. Dilators were passed sequentially through the rectum. The EEA device was then inserted and advanced through the rectal stump and the spike was deployed. The anvil was coupled to the stapler, which was fired after ensuring that the colon had a normal vertical lie without twist or tension.

**Figure 1:**
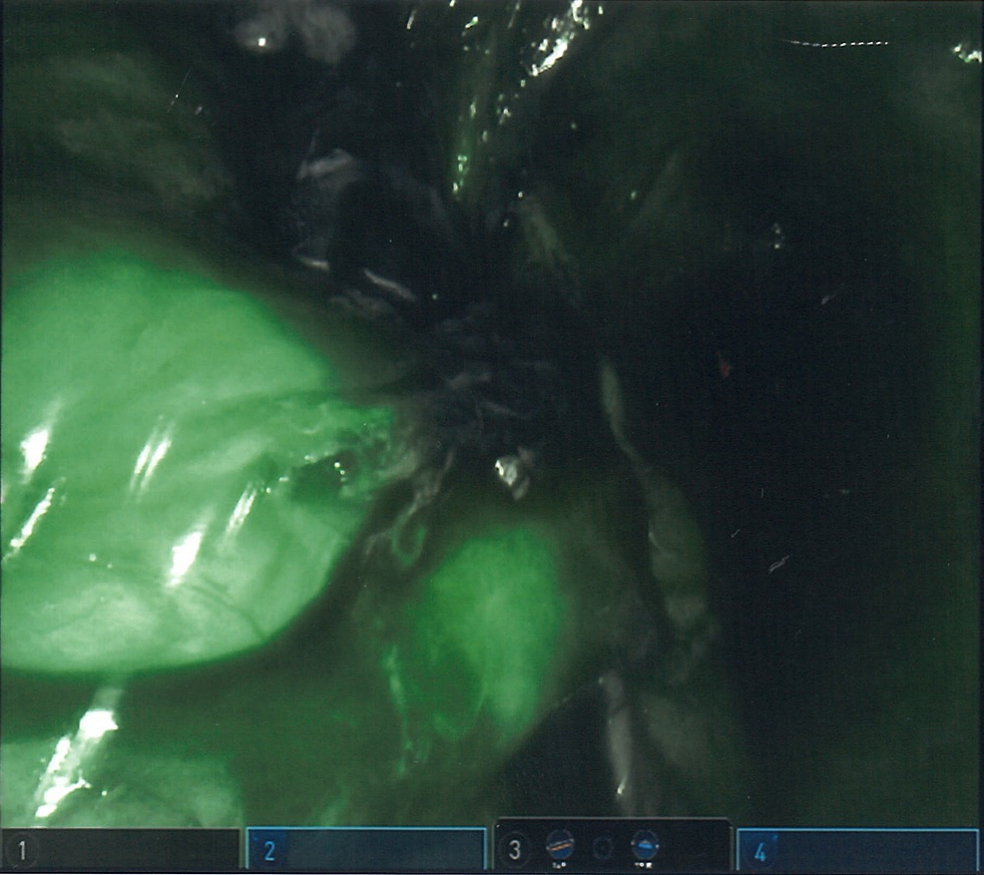
Intraoperative photo featuring ICG fluorescence imaging of colorectal anastomosis. ICG: indocyanine green

After completing the anastomosis, a 5 mg (2 mL) dose of ICG was administered through the patient’s left forearm IV catheter to evaluate perfusion to the anastomosis. However, no fluorescence was visualized. A second 5 mg (2 mL) dose of ICG was then administered via the patient’s right forearm IV and revealed robust perfusion to the anastomosis (Figure 1).

**Figure 2:**
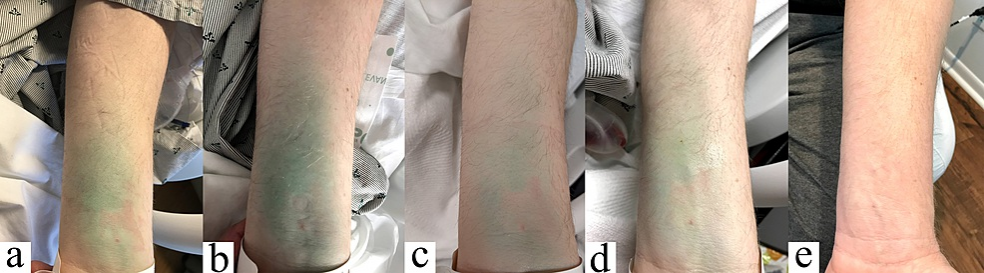
Progression of ICG infiltration from postoperative day one through postoperative day four (a-d), and complete resolution by postoperative day 18 (e). ICG: indocyanine green

On postoperative day one, the patient was noted to have a green discoloration along the volar aspect of his left forearm at the site of the IV catheter; infiltration of the initial dose of ICG was suspected. He denied discomfort at the site and did not have any sensory, circulatory, or motor deficits. He was treated with heat packs over the area of infiltration and left upper extremity elevation. The discoloration gradually abated, as demonstrated in successive photos in Figure 2, and the patient was discharged on postoperative day four. The patient was seen in the clinic on postoperative day 18 at which point the discoloration had resolved.

## Discussion

ICG is commonly used in abdominal surgery to aid in the identification of anatomy as well as to evaluate tissue perfusion. ICG instilled 15-45 minutes prior to cholecystectomy allows concentration in bile, whereas intestinal perfusion may be evaluated 2-3 minutes after ICG administration. Laparoscopic fluorescence imaging technology utilizes excitatory wavelengths and near-infrared imaging to visualize ICG fluorescence in blood vessels and/or biliary structures. Rare adverse effects of ICG administration have been reported, including anaphylaxis and death [8,9]. However, information regarding the incidence, treatment, and prognosis of subcutaneous ICG infiltration is not readily available. The lethal dose 50 of ICG is much higher than doses routinely used in laparoscopic/robotic surgery, and a repeat injection may be safely repeated via a different IV catheter [1].

A study regarding the use of ICG in sentinel lymph node biopsy (SLNB) in breast cancer surgery noted that ICG was approved for IV use; however, during SLNB, ICG is instilled intradermally and subcutaneously along the areola. In a study regarding the efficacy of ICG in SLNB, there were no adverse reactions to ICG, and there was no permanent staining of the skin among the 109 study participants [6].

It was speculated that the Trendelenburg position of the patient during the infiltration of the ICG contributed to the proximal spread of the ICG due to gravity. In the absence of a comparison of infiltration without Trendelenburg positioning, the effects of the patient’s position on the spread and migration of the ICG are unknown. However, the Starling equation shows that interstitial fluid formation is affected by hydrostatic pressure, porosity of the capillary walls, and the concentration of proteins in the blood and interstitium [10]. Given the theoretical decreased hydrostatic pressure in the arm in the Trendelenburg position, it is possible that less interstitial fluid was formed during the procedure, and with less dilution of the ICG in the interstitium, the staining may have been more pronounced than would be expected had the patient been supine. However, the increased lymphatic flow with gravity may have increased the lymphatic uptake of ICG and decreased the staining [11].

## Conclusions

In general, ICG is a safe diagnostic agent with low toxicity. Skin discoloration due to the subcutaneous infiltration of ICG resolves quickly. There are no reports of permanent skin tattooing when used for SLNB in breast surgery. Our experience has revealed that elevation and heat packs may facilitate lymphatic drainage and resolution of the discoloration. If ICG is injected and the expected fluorescence does not materialize, and a technological mishap has been excluded, infiltration should be suspected and the IV should be removed. Supportive care and anticipatory guidance may be provided for subcutaneously infiltrated ICG.
